# The Role of Platelet-Rich Fibrin (PRF) in the Prevention of Medication-Related Osteonecrosis of the Jaw (MRONJ)

**DOI:** 10.1155/2021/4948139

**Published:** 2021-05-15

**Authors:** Michele Miranda, Francesco Gianfreda, Carlo Raffone, Donato Antonacci, Valeria Pistilli, Patrizio Bollero

**Affiliations:** ^1^Department of Clinical Sciences and Translational Medicine, University of Rome “Tor Vergata”, 00133 Rome, Italy; ^2^Department of Industrial Engineering, University of Rome “Tor Vergata”, 00133 Rome, Italy; ^3^Independent Researcher, 00198 Rome, Italy; ^4^Independent Researcher, 70121 Bari, Italy; ^5^Department of System Medicine, University of Rome “Tor Vergata”, 00133 Rome, Italy

## Abstract

Dentoalveolar surgery is probably the major risk factor for MRONJ and for other complications following a tooth extraction, especially in patients affected by systemic diseases. The aim of this retrospective study is to evaluate whether a PRF plug inserted in the post extraction socket can prevent the onset of MRONJ. The patients were divided into two groups according to the surgical protocol that included the insertion or not of the PRF following the extraction and all the anamnestic, and clinical data were analyzed. In the control group, 5 patients developed MRONJ (19.23%) while in the study group, any case of MRONJ was reported. In the control group, patients who developed MRONJ had a CTX with less than 100 pg/mL (5 high-risk patients, Spearman's rank *r* = .547, *p* < .001). The use of platelet concentrates in patients with high risk of MRONJ is a user-friendly technique with an excellent cost-benefit ratio in oral surgery.

## 1. Introduction

The medication-related osteonecrosis of the jaw (MRONJ) is a term used to describe the complications that can occur in patients receiving specific antiresorptive or antiangiogenic drugs [1]. MRONJ is diagnosed when all the following pathological conditions are present: current or previous administration of antiresorptive or antiangiogenic agents, exposed bone or the bone that can be probed through an intraoral or extraoral fistula in the maxillofacial region that has persisted for longer than 8 weeks, and no history of radiation therapy of the jaws. However, for the “workshop of European task force on medication-related osteonecrosis of the jaw—current challenges”, the requirement of 8 weeks' observation of the potential manifestation of MRONJ to adapt to the case definition may no longer be necessary to make differential diagnosis with other bone and jaw diseases. About a third of half of the affected individuals currently develop MRONJ without a history of tooth extraction or other trauma [2]. The most important factor in the pathogenesis is the inhibition of the osteoclastic activity and the bone remodeling [3] caused by antiresorptive or antiangiogenic drugs. Other factors increasing the risk of MRONJ are the presence of the inflammation or infection interesting the oral site, the immune dysfunction [4], or some drug side effects such as the inhibition of angiogenesis and soft tissue toxicity [5, 6]. Two different variables are considered: the therapeutic indications, osteoporosis and osteopenia or malignancy, and the type of medication used, bisphosphonates (BPs) or other antiresorptive or antiangiogenic medications (non-BPs). Several studies show that the duration of the therapy is an important risk factor when considering patients with cancer exposed to zoledronate or denosumab. Dentoalveolar surgery is probably the major risk factor for MRONJ and for other complications following a tooth extraction, especially in patients affected by systemic diseases [7, 8]. PRF has been used for regenerative procedures in various fields of medicine, including dentistry and reconstructive surgery in order to deliver high concentrations of autologous growth factors directly to wounds [9]. These growth factors have been shown to be chemotactic for various cell types, such as stem cells and fibroblasts, creating tissue microenvironments and directly influencing the proliferation and differentiation of progenitor cells [10]. What is more, the fibrin network of PRF, thanks to leukocytes present inside, can fight infections in unhealed wounds thus improving clinical outcomes [11]. Osteomyelitis is reported in 9.5% of wisdom tooth removal and when a PRF plug was inserted after extraction, this was significantly reduced to 1% of cases [12]. Histopathological parameters of chronic/suppurative osteomyelitis, medication-related osteonecrosis of the jaw (MRONJ), and osteoradionecrosis (ORN) are the same, and for this reason, the use of PRF could be a protective factor in the prevention of MRONJ after dental extractions [13]. Şahin et al. [14] assessed that the use of PRF seems to be a good alternative for prevention of MRONJ, promoting a high-rate success of surgery and improving healing with better final results. On the other hand, systematic reviews showed that there is still an insufficient evidence on the real benefits of the platelet concentrates in order to improve healing or prevent ONJ lesions [15, 16]. A clinical trial with a group treated without platelet concentrates seems challenging and unethical since there is not a gold standard treatment for MRONJ. The aim of this retrospective study is to investigate, through operator registers of the Department of “Special Needs Dentistry with Protected Paths” at the Tor Vergata University Policlinic in Rome (Director Prof. Patrizio Bollero), if patients taking antiresorptive or antiangiogenic agents can lead to the same prevalence of MRONJ after dental extractions depending on the use or not of PRF.

## 2. Materials and Methods

This retrospective study has been conducted in accordance with the requirements of the Helsinki Declaration of 1975 and the patient provided the written informed consent in the Department of “Special Needs Dentistry with Protected Paths” at the Tor Vergata University Policlinic in Rome (Director Prof. Patrizio Bollero).

The ethical approval was obtained from the Independent Ethics Committee of the University of “Roma Tor Vergata” (experimentation register RS 59.21).

Thirty-seven patients taking antiresorptive or antiangiogenic agents (36 female and 1 male) with ages ranging from 54 to 91 whose extractions were deemed urgent and could not be postponed were studied using a private register. The studied patients were divided in two groups: the first group of 26 patients taking antiresorptive or antiangiogenic agents (control group), treated from January 2015 to July 2016, had dental extractions trying to be as less invasive as possible to avoid cases of osteonecrosis.

Subsequently, following an interdisciplinary comparison with hematologists, a new method was proposed from September 2016 in which the classic extractions were accompanied to the use of the PRF. From September 2016 to December 2018, 11 patients were treated with dental extraction and a PRF plug in addition (study group). All the patients included gave informed consent and were informed about the possible complications of dental extraction under medication treatment.

Inclusion criteria were (1) administration of oral bisphosphonates or antiresorptive/antiangiogenic drugs, for at least 12 months and (2) no clinical signs of MRONJ during the first visit.

Exclusion criteria were (1) tooth extraction or other oral surgery treatments in the 3 months before the study, (2) pregnant or breast-feeding women, and (3) hypersensitivity to any medication used.

### 2.1. Technical Procedures

Before starting the surgical procedures, all the subjects underwent dental panoramic radiography and two weeks before tooth extraction, patients underwent periodontal procedures, in particular root scaling to establish adequate oral hygiene conditions, and the oral hygiene instructions were given. The antimicrobial prophylaxis was performed using amoxicillin and clavulanic acid (1 g every 12 hours, 5 days before and 5 days after dental treatment) and metronidazole (250 mg every 12 hours, 2 days before and 2 days after dental treatment). In the case of penicillin's allergy, azithromycin was used (500 mg, 2 hours before dental treatment and 1 tablet per day for 5 days after surgery). Dental nerve anesthesia was achieved using 3% mepivacaine hydrochloride and epinephrine 1 : 100,000. Dental extraction was followed by delicate curettage. In the control group, after the dental extraction and the curettage, a 4-0 x-suture with monofilament polyamide was done without making any flap. The x-suture had exclusively a hemostatic function. In the experimental group, PRF was obtained from the patient's peripheral venous blood and it was centrifuged at 3000 rpm for 10 minutes in 10 ml tubes without anticoagulant. The PRF was then inserted into the postextraction sockets and then a 4-0 x-suture with monofilament polyamide was done. Like the control group, no flaps were performed in this case as well. Patients were given standard postoperative instructions and advised not to brush in the treated area until sutures were removed, written oral hygiene and postoperative instructions were then given to all of the patients. Suturing was removed after 7 days and the monitoring was carried out at 1, 2, 3, and 6 months. Patients were trained to avoid wearing removable dentures for 9 months and to recognize signs and symptoms of MRONJ.

### 2.2. Statistical Analysis

During the first examination, medical records were collected (Table 1). Particular attention was given to the medical history, type of disease (osteometabolic or oncological disease), pharmacological therapy, duration of therapy and chemotherapy/radiotherapy or corticosteroids therapy. CTX serum (beta-CrossLaps) levels were analyzed to establish risk class for every patient. What is more, manifestations of MRONJ, signs and symptoms were collected in a register. Statistical analysis was done using SPSS (IBM Corporation) software. Data were analyzed in order to evaluate correlations and differences of the compared groups.

## 3. Results

**Figure 1:**
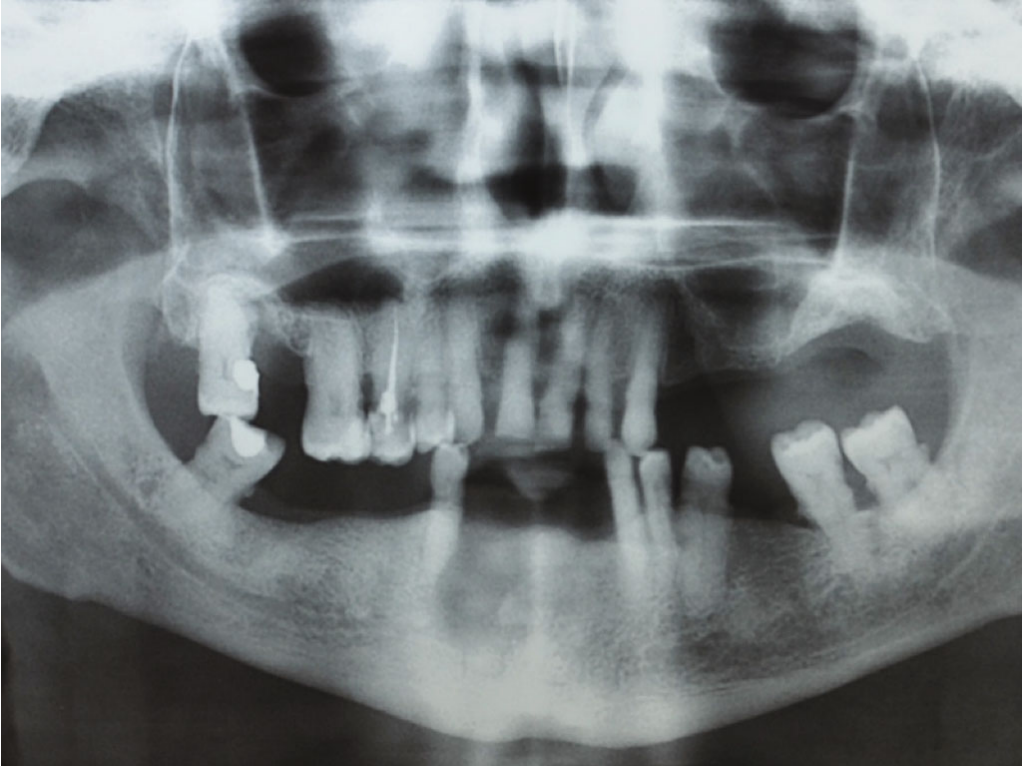
Radiographic signs of MRONJ.

**Figure 2:**
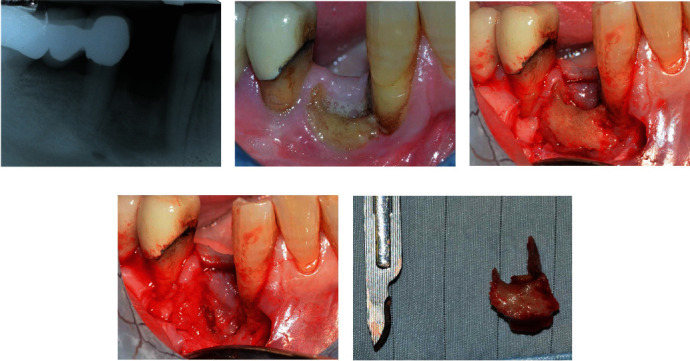
Clinical and radiological images showing examples of a MRONJ stage 2. A female patient with 55 years old in treatment with endovenous ibandronate every 3 months for oncological disease (69 months therapy). MRONJ manifestation after 4 months from the extraction of a single tooth. High risk due to low CTX level (less than 100 pg/mL). Radiographic (a) and clinical images (b) showing MRONJ four months after extraction (control group). Exposed bone, pain, swelling, and ongoing infection can be detected. From the radiological point of view, it is possible to observe a radiolucency with undefined margins in correspondence with the postextraction socket. Once the trapezoidal access flap has been performed, it is possible to observe an important quantity of necrotic bone (c). A curettage of the site was performed after the complete removal of the necrotic bone (d). The exported lesion (e) is larger than that which could be observed radiographically. Careful patient education and a meticulous follow-up system is important in these types of patients to intercept MRONJ in the early stages. In the specific case, a progression of the lesion could have affected the periodontal support of the adjacent teeth and affected the basal bone of the mandible and the mental nerve.

Thirty-seven patients took part in this study, 36 (96.2%) of whom were women. The mean age at presentation was 70.69 (SD = 8.03) years for the control group and 74.81 (SD = 8.88) for the study group. In total, 19 patients were in treatment for osteometabolic disease and 18 for oncological disease. In the control group with 22 patients (84.59%), BFs were administered orally and 2 (7.69%) patients were treated with intravenous BFs and 2 (7.69%) with denosumab. In the test group, all the patients, except one in treatment with denosumab, used the oral route of administration. Four patients in the control group received concomitant systemic chemotherapy (2 patients received cyclophosphamide and other 2 patients received 5-fluorouracil), and one patient received steroids in the study group. In total, 69 extractions were performed. Regarding the control group, 42 extractions were carried out, 15 (35.71%) from the maxilla and 27 (64.29%) from the mandible. In the study group, 27 extractions were performed, 9 (33.33%) from the maxilla and 18 (66.66%) from the mandible. The average numbers of teeth extracted for each patient in the control group were 1.61 (SD = .75) and 2.45 (SD = 1.63) in the study group. In the control group, 5 patients developed MRONJ (19.23%) (Figures 1 and 2 and Table 2), while in the study group, any case of MRONJ was reported (Table 1). Significant differences were not found regarding MRONJ manifestations between groups (*X*^2^ = 2.446, *p* = .118). Chi-square test can be affected by sample size. In the control group, patients who developed MRONJ had a CTX with less than 100 pg/mL (5 high-risk patients, Spearman's rank *r* = .547, *p* < .001) and all extractions with this complication were in the jaw (3 in anterior mandible, 2 in posterior mandible); 2 of MRONJ patients were in cure for an osteometabolic disease and 3 for oncological reasons. The mean time of MRONJ manifestation from extraction was of 3.6 months (SD ± 1.14). MRONJ was stage 2 in all the cases. Two of MRONJ patients have additional risk factor (1 smoking habits and 1 diabetes) and 3 patients were in treatment with oral BFs and 2 with endovenous BFs.

## 4. Discussion

Despite that MRONJ may develop spontaneously, up to 80% of the cases are related to dental extraction or interventions leading to bone exposure [17]. A multidisciplinary approach to the treatment of patients taking antiresorptive or antiangiogenic medications is definitely needed. Many strategies for the prevention of MRONJ have been evaluated but authors are still searching for consensus. For instance, the concept of drug holiday is still controversial. In a 2011 summary document on the long-term safety of BP therapy for osteoporosis, the FDA stated that “there was no substantial data available to guide decision regarding the initiation or duration of a drug holiday” [18]. Although, in 2014, a special committee of the American Association of Oral and Maxilofacial Surgeon [19] asserted that the modified drug holiday strategy by Damm and Jones [20] is a prudent approach for patients with extended exposure histories (>4 yr). In our study, due to limited data, drug holiday was not included. During the clinical trials, the authors choose to investigate about the usefulness of using a systemic marker of bone turnover to assess the risk of MRONJ. The “American Association of Oral and Maxillofacial Surgeon position paper on medication related osteonecrosis of the jaw—2014 Update” [19] stated that this method has not been validated by literature but research must be performed. In our study, all the patients that developed MRONJ had a CTX value below 150 pg/mL with extraction performed in mandible.

Several strategies have been proposed to prevent the onset of MRONJ. The use of Nd : YAG and alveoloplasty with piezoelectric instruments and the use of growth factors seem to be tools to enhance healing in these patients [20]. A study by Mozzati et. al demonstrated on a sample of 1480 extractions that there are no significant differences in the healing of alveoli sutured by the first or second intention and the manifestation of MRONJ [21].

The number of teeth extracted does not seem important in the development of these lesions (Table 2). The BPs, thanks to the structure with a carbon atom in the center of two phosphate groups, turn out to be very stable molecules thanks to their resistance to enzymatic hydrolysis and their ability to bind divalent metal ions, such as Ca2+ [22]. It is believed that the antiresorption potency may be linked to the inhibition of the enzyme farnesyl pyrophosphate synthase (FPPS) and their ability to bind to hydroxyapatite [23]. Osteoclasts, in the phases in which they initiate bone remodeling, are in contact with a strongly acidic environment which facilitates the release of bisphosphonates from the bone surface, leading to an increase in the local concentrations of the same molecule [24]. The bisphosphonate then enters the metabolism of the osteoclast by inhibiting its activity due to its toxic effect which leads to a fragmentation of the cell with consequent apoptosis [25]. Furthermore, bisphosphonates can inhibit the intracellular transport mechanisms of the osteoclast leading to a disorganization of the actin cytoskeleton and to the loss of actin rings or to disturbances in the formation of the ruffled edge of the osteoclasts [26].

Bisphosphonates are also absorbed by osteoblasts, macrophages, epithelial and endothelial cells, circulating monocytes, and neoplastic cells, such as myeloma and prostate cancer cells [27]. In mice treated with pamidronate (Pam), tooth extraction followed by oral infection with *Fusobacterium nucleatum* caused BONJ-like lesions and delayed epithelial healing. In both in vitro and in vivo experiments, the combination of Pam and *Fusobacterium nucleatum* caused the death of gingival fibroblasts (GF) and downregulated their production of the keratinocyte growth factor (KGF), which induces growth and migration of epithelial cells [28]. Nitrogen-containing BPs showed direct inflammatory or necrotic effects on soft tissues often increased by the presence of the lipopolysaccharide typical of periodontopathogenic bacteria [29].

PRF, containing white blood cells in combination with neutrophils and platelets, is able to enhance tissue wound healing, improve angiogenesis, and enhance tissue formation. In particular, the peculiarity of PRF consists in acting as a scaffolding material in which leukocytes, macrophages, neutrophils, and platelets present in supraphysiological doses release chemotactic factors and growth factors for wound healing. In addition, fibrin, by trapping platelets, acts as a reservoir for growth factors that are released over time from 10 to 14 days [30]. The importance of leukocytes on their key role, both for their anti-infective action and for immune regulation especially in preventing the contamination of the lesions by Gram-negative bacteria and related toxins also responsible for common osteomyelitis in the oral cavity, should also be emphasized. According to a study by Clipet et al. [31], PRF induces the survival and proliferation of fibroblast and keratinocytes and this aspect could play a very important role in antagonizing the effects of bisphosphonates especially where there is bacterial overinfection. Finally, PRF was chosen by practitioners to prevent MRONJ as it has a high concentration of PDGF and is an essential regulator for the migration, proliferation, and survival of mesenchymal cell lines, TGF-*β*1, the most commonly released growth factor in autologous bone and which induces a massive synthesis of type 1 collagen and fibronectin and VEGF, the most potent growth factor leading to tissue angiogenesis [32–34]. The first week after dental extraction is the most critical period because the mechanism of action of BPs leads to a decrease of oral epithelial cell migration, increased apoptosis, and inhibition of osteoclastic activity [33], so the use of PRF can improve the outcome of wound healing preventing osteonecrosis and leading to an early epithelization [35, 36]. All the patients of the study treated with PRF underwent to a good and fast postoperative healing and improved bone repair confirming the hypotheses of the literature [14–30]. In a systematic review of Fortunato et al. [37], out of a total of 1219 dental extractions recorded with autologous platelet concentrates for prevention of osteonecrosis, only 12 cases of MRONJ have been reported (1%) in patients with a history of high-dose antiresorptive treatment. So, the data of this research seems to validate the important role of PRF in the prevention of MRONJ, specially in patients at high risk.

The data in the literature regarding the incidence and prevalence of MRONJ are discordant. Some studies report a reported incidence of MRONJ after tooth extraction which is estimated at 2.9% in cancer patients and 0.15% in patients being treated for osteoporosis [38]. Other more recent studies instead show that the incidence can be 18.6% in relation to the dose and time of administration of bisphosphonates in cancer patients [39, 40].

In our study, a slightly higher incidence of ONJ was found in treated patients in the control group (19.23%). Significant differences were not found regarding MRONJ manifestations between groups (*X*^2^ = 2.446, *p* = .118). Chi-square test can be affected by the sample size. This may be due to multiple factors such as the small sample of cases, the difference in patient therapy, route, and time of administrations of drugs and low CTX levels in patients at increased risk. Furthermore, MRONJ was associated with higher risk CTX (Spearman's rank *r* = .547, *p* < .001) and all extractions with this complication were in the mandible and in stage 2. The limitations of the study are represented by the inhomogeneity and the small number of samples which allowed us to carry out a purely descriptive statistic. Future studies will be needed to understand whether PRF may be the keystone for the surgical treatment of patients on bisphosphonates.

## 5. Conclusions

The use of platelet concentrates with the aim of preventing the onset of MRONJ is a user-friendly technique with an excellent cost-benefit ratio. The main limitation of our study is the low number of cases analyzed retrospectively. In order to have more specific data and being the PRF use a safe and healing-enhancing practice, dentists should be encouraged to use platelet derivatives in all cases where normal wound healing can be impaired, especially in cases where there is a risk of osteonecrosis and osteomyelitis.

## Figures and Tables

**Table 1:** General information of the study.

Variable	Control group		Study group	
	*N*	%	*N*	%
Gender				
Male	1	3.85%	0	0%
Female	25	96.15%	11	100%
Mean age (years)	70.69 (SD = 8.03)		74.81 (SD = 8.88)	
Type of medication used				
Alendronate	12	46.15%	7	63.64%
Risedronate	5	19.23%	1	9.09%
Ibandronate	2	7.69%	0	0%
Denosumab	2	7.69%	1	9.09%
Alendronate + cyclophosphamide	2	7.69%	0	0%
Alendronate + 5 − fluorouracil	2	7.69%	0	0%
Alendronate + steroids	1	3.85%	2	18.18%
Type of disease				
Osteoporosis	14	53.85%	5	45.45%
Oncological disease	12	46.15%	6	54.55%
Route of drug therapy				
Oral	22	84.59%	10	90.91%
Intravenous	2	7.69%	0	0%
Subcutaneous	2	7.69%	1	9.09%
Mean duration of drug therapy (months)				
Oral	44.18 (SD = 21.18)		59.20 (SD = 14.92)	
Intravenous	56 (SD = 18.38)		—	
Subcutaneous	49 (SD = 18.38)		37	
Risk level associated to CTX value				
High risk CTX level less than 100 pg/mL	6	23.07%	4	36.36%
Moderate risk CTX level between 100 and 150 pg/mL	5	19.23%	3	27.27%
Low risk CTX level above 150 pg/mL	15	57.69%	4	36.36%
Tooth extracted				
Mandible	27	64.28%	18	66.67%
Maxilla	15	35.71%	9	33.33%
Reason for extraction				
Periodontitis	8	19.04%	7	25.9%
Destructive tooth decay	11	26.19%	11	40.7%
Residual roots	23	54.76%	9	33.3%
Other risk factors				
Diabetes	4	15.38%	1	33.33%
Smoking habits	5	19.23%	2	66.67%

**Table 2:** Clinical information of patients presenting MRONJ.

Data collected from MRONJ manifestations					
Patients	#1	#2	#3	#4	#5
Age (years)	66	75	68	55	81
Gender	F	F	F	F	F
Risk factors	Smoking habits	x	Diabetes	x	x
Type of systemic disease	Osteoporosis	Oncological disease	Oncological disease	Oncological disease	Osteoporosis
Medication	Alendronate	Ibandronate	Alendronate + 5 − fluorouracil	Ibandronate	Alendronate
Route of drug therapy	Oral	Intravenous	Oral	Intravenous	Oral
Duration of drug therapy (months)	88	43	62	69	55
Risk associated to CTX	High	High	High	High	High
MRONJ stage	2	2	2	2	2
MRONJ site	Anterior mandible	Anterior mandible	Posterior mandible	Posterior mandible	Anterior mandible
Number of tooth extracted	3	1	2	1	2
Manifestation of MRONJ after extraction (months)	2	4	3	4	5

## Data Availability

The medical history, type of disease, pharmacological therapy, duration of therapy and chemotherapy/radiotherapy or corticosteroids therapy, CTX serum (beta-CrossLaps) levels, manifestations of MRONJ, MRONJ signs, and MRONJ stage data used to support the findings of this study are included within the article.
